# Planar binary-phase lens for super-oscillatory optical hollow needles

**DOI:** 10.1038/s41598-017-05060-2

**Published:** 2017-07-05

**Authors:** Gang Chen, Zhixiang Wu, Anping Yu, Kun Zhang, Jing Wu, Luru Dai, Zhongquan Wen, Yinghu He, Zhihai Zhang, Senlin Jiang, Changtao Wang, Xiangang Luo

**Affiliations:** 1 Key Laboratory of Optoelectronic Technology and Systems (Chongqing University), Ministry of Education, and Key Disciplines Lab of Novel Micro-nano Devices and System Technology, Chongqing University, 173 Shazheng Street, Shapingba, Chongqing 400044 China; 2 National Center for Nanoscience and Technology, No.11 Zhong Guan CunBei Yi Tiao, Beijing, 100190 China; 3 State Key Laboratory of Optical Technologies on Nano-Fabrication and Micro-Engineering, Institute of Optics and Electronics, Chinese Academy of Science, P. R. Box 350, Chengdu, 610209 China; 4 Department of Physics, Zunyi Normal College, Zunyi, 563006 China

## Abstract

Optical hollow beams are suitable for materials processing, optical micromanipulation, microscopy, and optical lithography. However, conventional optical hollow beams are diffraction-limited. The generation of sub-wavelength optical hollow beams using a high numerical aperture objective lens and pupil filters has been theoretically proposed. Although sub-diffraction hollow spot has been reported, nondiffracting hollow beams of sub-diffraction transverse dimensions have not yet been experimentally demonstrated. Here, a planar lens based on binary-phase modulation is proposed to overcome these constraints. The lens has an ultra-long focal length of 300*λ*. An azimuthally polarized optical hollow needle is experimentally demonstrated with a super-oscillatory transverse size (less than 0.38*λ*/NA) of 0.34*λ* to 0.42*λ*, where *λ* is the working wavelength and NA is the lens numerical aperture, and a large depth of focus of 6.5*λ*. For a sub-diffraction transverse size of 0.34*λ* to 0.52*λ*, the nondiffracting propagation distance of the proposed optical hollow needle is greater than 10*λ*. Numerical simulation also reveals a good penetrability of the proposed optical hollow needle at an air-water interface, where the needle propagates through water with a doubled propagation distance and without loss of its super-oscillatory property. The proposed lens is suitable for nanofabrication, optical nanomanipulation, super-resolution imaging, and nanolithography applications.

## Introduction

Nondiffracting beams are propagating waves, which can travel without divergence over a comparative long distance. A nondiffracting beam was firstly proposed theoretically^1^ and then experimentally demonstrated by Durnin *et al*.^2^ in 1987 using a zero-order Bessel beam created by focusing a plane-wave-illuminated circular slit with a conventional positive lens. Nondiffracting beams have a number of unique properties, such as nondiffracting propagation, a highly-localized intensity distribution, and beam self-reconstruction, and have therefore generated wide theoretical and experimental interest. A variety of methods have been proposed for the generation of nondiffracting beams. In addition to the method proposed by Durnin *et al*., other commonly used approaches include holographic methods^3, 4^, an axicon lens^5^, a cemented doublet lens^6^, a spatial-light-modulator (SLM)^7, 8^, and a digital micro-mirror device^9^. Nondiffracting beams have been successfully applied in materials processing^10^ and optical research^11^. Due to their self-reconstructing property, Bessel beams have shown great advantage in optical microscopy by increasing image quality and the penetration depth in dense media^12^, and have demonstrated unexpected robustness against deflection and a reduction in scattering artifacts. Bessel beams have also been utilized in nanolithography^13^. However, conventionally generated nondiffracting beams are restricted by the diffraction-limit 0.5*λ*/NA, where *λ* is the working wavelength and NA is the optical numerical aperture. To further improve the optical resolution, it would be attractive to a method of creating nondiffracting beams with sub-diffraction transverse dimensions. The generation of sub-wavelength^14^ and sub-diffraction^15^ optical needles has also been theoretically studied. Optical super-oscillation is referred to the optical phenomenon, in which the local spatial frequency of the optical field is larger than its global spatial frequency, and theoretically, it provides a possible means of creating arbitrary small features in far-field optics with the superposition of band-limited functions^16, 17^. Based on this concept, sub-diffraction focusing has been demonstrated^18–24^. The theoretical design of sub-diffraction optical needles was also proposed based on super-oscillatory planar lenses^25^. Recently, sub-diffraction optical needles with transverse polarization have been reported for visible^26, 27^ and violet light^28^. The generation of a sub-diffraction longitudinally polarized optical needle with a propagation distance of 4*λ* was firstly proposed theoretically by focusing a radially polarized Bessel-Gaussian beam using a combination of a binary-phase optical element and a high-NA lens^29^. The experimental generation of such a sub-diffraction optical needle was demonstrated recently by focusing radially polarized light with a single planar binary-phase lens^30^.

Among possible nondiffracting beam candidates, hollow beams have a zero intensity along the beam center-axis, making them promising for applications in optical micromanipulation, microscopy, and lithography, and have been widely investigated^31–35^. A hollow Bessel beam was generated by focusing a vortex beam with a water-immersed objective lens, and was applied to improve the performance of stimulated emission depletion (STED) microscopy^36^. A nondiffracting beam with sub-wavelength features has been theoretically investigated in far-field propagation^37^. The generation of sub-diffraction hollow beams also has been theoretically proposed using a high-NA objective lens and a six-zone complex pupil filter^38^. In the present work, an ultra-long super-oscillatory optical hollow needle is demonstrated by focusing an azimuthally polarized wave using a single planar binary-phase lens with an NA of 0.908. Experimental investigation reveals the formation of a 6.5*λ*-long azimuthally polarized optical hollow needle with a transverse full width at half maximum (FWHM) less than the super-oscillatory criterion 0.38*λ*/NA^39^. Its long propagation distance and super-oscillatory transverse size make such hollow beam distinct from the previously reported sub-diffraction hollow spot (with FWHM less than 0.5*λ*/NA)^40^. It is also revealed that the optical hollow needle propagates for a length of 10*λ* with a transverse FWHM less than the diffraction limit 0.5*λ*/NA. Numerical simulation shows that the proposed optical hollow needle has good penetrability at an air-water interface with a substantially enlarged needle length in water.

## Materials and Methods

### Binary-phase lens design

Although continuous phase modulation is favorable in super-oscillatory lens design^20^, a binary-phase lens is much easier to fabricate with comparatively low cost. Therefore, a planar lens based on binary-phase modulation was designed for a normally incident wave with azimuthal polarization at *λ* = 632.8 nm to realize a sub-diffraction and super-oscillatory optical hollow needle. Similar to a previously reported binary-phase lens design^24, 30, 40^, the lens designed here consists of a series of concentric dielectric ring belts with a relative phase delay of π. The widths of the ring belts are integer multiples of 400 nm. The electrical field distribution of the incident wave is Laguerre-Gaussian without angular momentum, which can be described according to a radial coordinate *r* and a height coordinate *z* in the spatial domain as

1 $$E(r,z)={E}_{0}\frac{{w}_{0}}{w{(z)}^{2}}r\,\exp (\frac{-{r}^{2}}{w{(z)}^{2}})\exp \{j[kz+\frac{k{r}^{2}}{2R(z)}-2\,\arctan (\frac{z}{{z}_{0}})]\},$$

where *E*_0_ is the incident electrical field amplitude, *w*_0_ is the beam waist size, *k* = 2π*/λ* is the wavenumber, *z*_0_ = π*w*_0_^2^/*λ* is the Rayleigh range, *w*(*z*) = *w*_0_[1 + (*z*/*z*_0_)^2^]^1/2^ is the beam width and *R*(*z*) = *z*[1 + (*z*_0_/*z*)^2^] is the radius of curvature at *z*. On the lens input surface, the transverse beam amplitude profile is defined by *w*_0_ = 331 μm and *z* = 276 mm. The diffraction electrical field can be obtained using the vectorial angular spectrum method^41^. For a circularly symmetrical planar optical device under the illumination of a co-axis incident wave with an azimuthal polarization direction *φ* and a circularly symmetrical distribution of amplitude and phase, the diffracted electrical field has only an azimuthal component, and can be calculated as

2 $$\{\begin{array}{rcl}{E}_{\varphi }(r,z) & = & {\int }_{0}^{\infty }A(\rho )\,\exp [j2{\rm{\pi }}q(\rho )z]{J}_{1}(2{\rm{\pi }}\rho r)2{\rm{\pi }}\rho {\rm{d}}\rho \\ A(\rho ) & = & {\int }_{0}^{\infty }g(r)t(r){J}_{1}(2{\rm{\pi }}\rho r)2{\rm{\pi }}r{\rm{d}}r\end{array},$$

where *ρ* is a radial coordinate in the frequency domain, *q*(*ρ*) = ((1/*λ* ^2^) − *ρ* ^2^)^1/2^, *z* is the wave propagation distance, *J*_1_ is the first-order Bessel function, and *g*(*r*) and *t*(*r*) are the electrical field distribution and lens transmittance function of the incident beam, respectively. The form of Eq. (2) is similar to that for scalar field diffraction, which employs the zero-order Bessel function *J*_0_ as the integral kernel instead.

**Figure 1:**
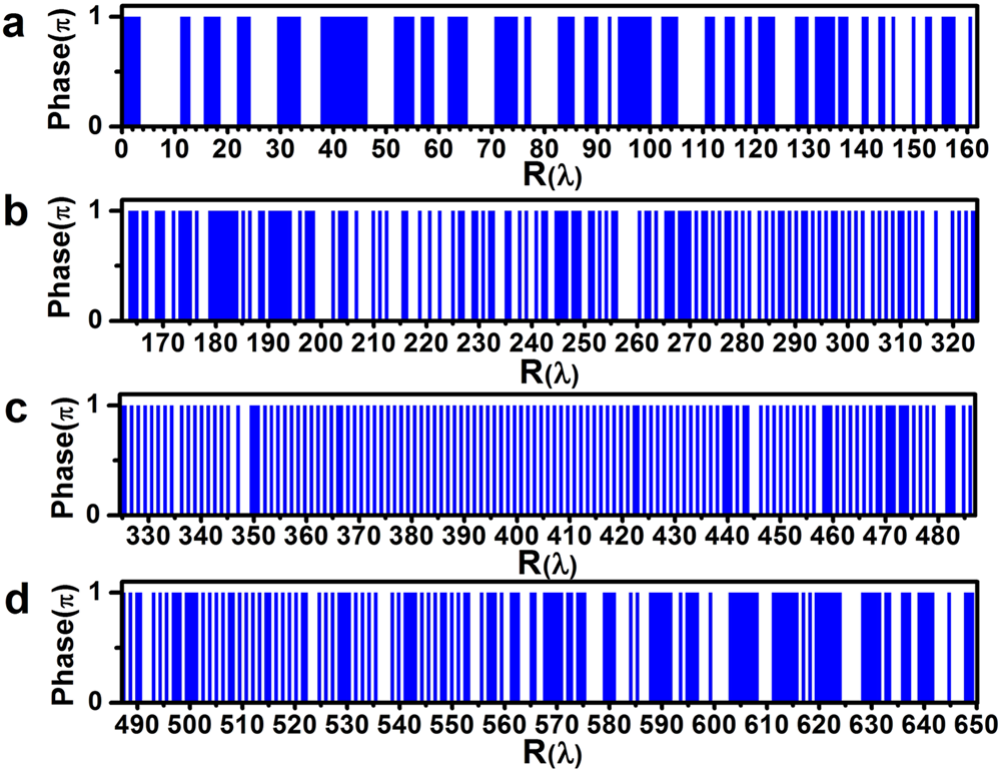
The optimized binary phase (i.e., 0 and π) distribution of the super-oscillatory lens with radius of 650*λ* for wavelength *λ* = 632.8 nm, where R is the radial coordinate and the rectangle blocks give the corresponding position and width of the 292 ring belts with phase of π. The ring belts located between two neighboring rectangle blocks have phase of 0.

**Figure 2:**
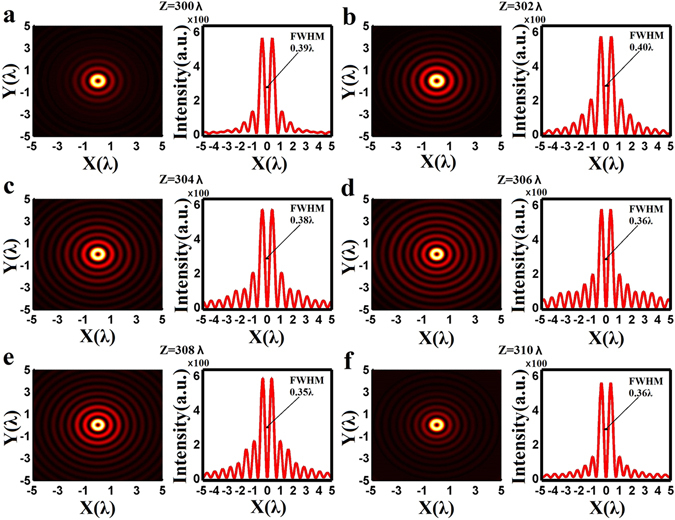
(**a**)**–**(**f**) The 2-dimentional color maps of the optical intensity distribution in the XY-plane and the optical intensity distribution curves along the x-axis obtained from Eq. (2) using the vectorial angular spectrum method at different propagation distances of 300*λ*, 302*λ*, 304*λ*, 306*λ*, 308*λ*, and 310*λ*, respectively, where the intensity is plotted in arbitrary unit.

**Figure 3:**
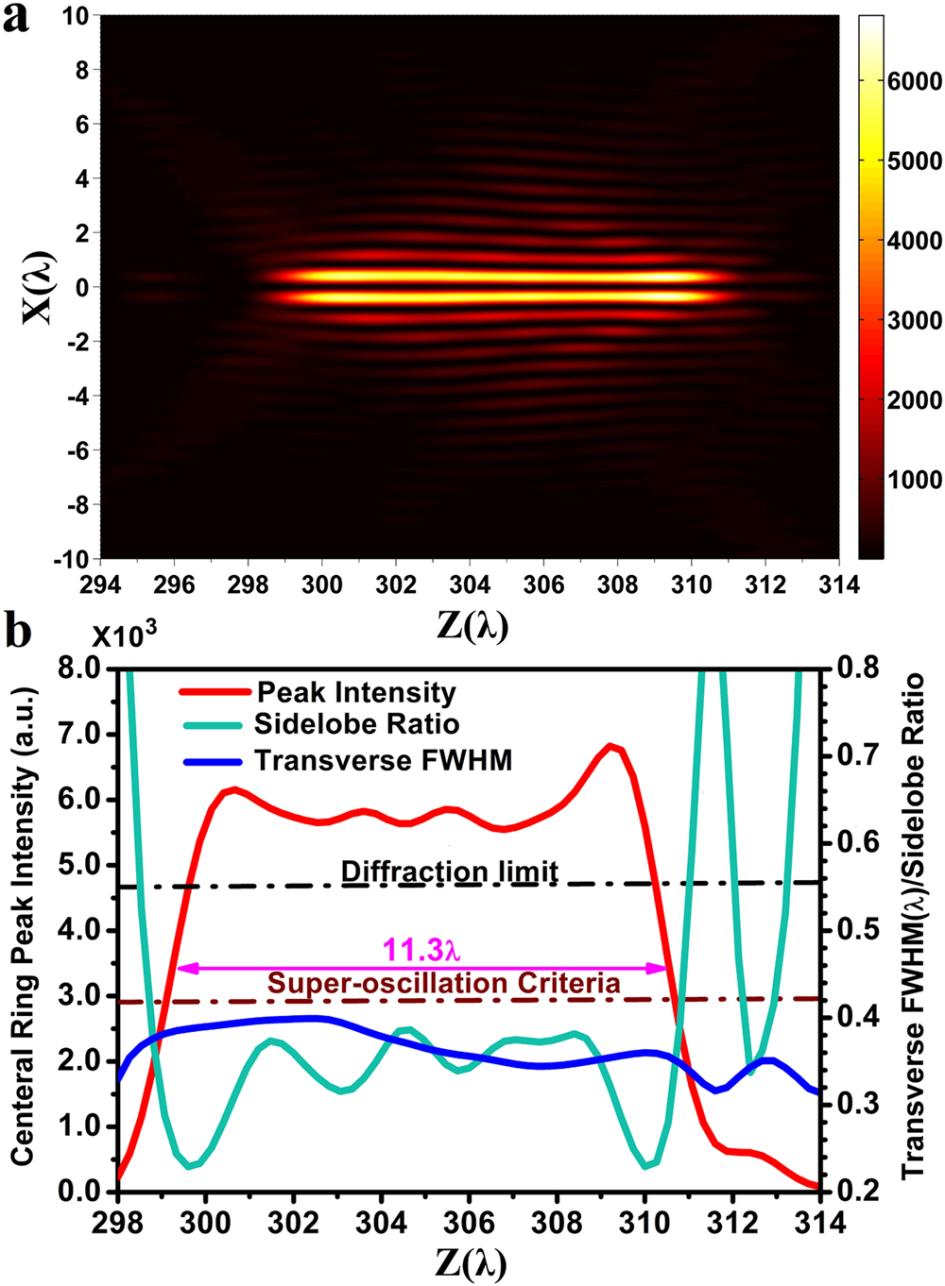
(**a**) The optical intensity distribution in the XZ propagation plane obtained from Eq. (2) using the vectorial angular spectrum method, where the intensity is plotted in arbitrary unit. (**b**) The theoretical central ring peak intensity (red), the transverse FWHM (blue), and sidelobe ratio (green) distributions along the propagation direction, where the black and wine-colored dashed-dotted lines indicate the diffraction limit and super-oscillatory criterion, respectively.

The lens has a radius of *R*_*lens*_ = 650*λ*, focal length *f* = 300*λ*, and an NA given as sin[arctan(*f*/*R*_*lens*_)] = 0.908. The corresponding diffraction limit is 0.551*λ* (i.e., 0.5*λ*/NA) and the super-oscillation criterion is 0.419*λ* (i.e., 0.38*λ*/NA). The binary-phase distribution of the lens was optimized using particle swarm optimization^42^ in conjunction with Eq. (2) to ensure that the diffracted electrical field forms a super-oscillatory optical hollow needle with a maximum transverse FWHM of the inner ring less than 0.4*λ* between *z* = 300*λ* and *z* = 310*λ* along the optic axis. The optimized lens phase distribution is presented along the radial direction in units of *λ* in Fig. 1. The ring belts with phase of π are depicted as rectangle blocks, which give their corresponding position and width. The ring belts with phase of 0 are located in the areas between two neighboring rectangle blocks. It is found that lens consists of 292 ring belts with phase of π. Figure 1 clearly indicates that most of the inner and outer ring belts have widths greater than a single increment of *λ*, compared with those 400 nm-width sub-wavelength ring belts clustered in the middle section at around 400*λ* (the Supplementary Information lists the radius and width of the ring belts with phase change of π). Figure 2(a)–(f) illustrate the 2-dimentional transverse optical intensity distributions on the XY plane calculated using Eq. (2) along the *z*-axis at *z* = 300*λ*, *z* = 302*λ*, *z* = 304*λ*, *z* = 306*λ*, *z* = 308*λ*, and *z* = 310*λ*, where the intensity is given in arbitrary unit. The optical intensity along the x-axis is also plotted for each of above cases, which clearly shows a strong central ring surrounded by several small sidelobe rings with gradually reducing intensity. As indicated by the black lines, the corresponding transverse FWHM values of the inner rings are found to be 0.39*λ*, 0.40*λ*, 0.38*λ*, 0.36*λ*, 0.35*λ*, and 0.36*λ*, respectively. As expected, all those FWHM values are smaller than 0.40*λ*, which breaks not only the traditional diffraction limit 0.551λ (i.e., 0.5λ/NA), but also the super-oscillation criterion 0.419λ (i.e., 0.38λ/NA). At those six positions, the difference in the peak intensity is less than 30%. To investigate the propagation property of the optical hollow needle, the calculated optical intensity distribution in the XZ propagation plane is depicted in Fig. 3(a) for the range *z* = 294*λ* to *z* = 314*λ*, which clearly shows a hollow needle structure between *z* = 299*λ* and *z* = 312*λ*. The transverse FWHM, the ratio of the maximum sidelobe intensity to the central lobe peak intensity (i.e., the sidelobe ratio), and the central ring peak intensity are plotted with respect to the propagation distance from *z* = 298*λ* to *z* = 314*λ* in Fig. 3(b). This data shows that the hollow needle has a flat top with a small intensity fluctuation, and the longitudinal FWHM of the hollow needle is about 11.3*λ*, which extends from *z* = 299.2*λ* to *z* = 310.5*λ*, as indicated by the magenta-colored arrow. In this region, the transverse FWHM is less than 0.4*λ*, and the sidelobe ratio is less than 38.4%. The minimum transverse FWHM is found to be 0.34*λ* at *z* = 307.6*λ*. The FWHM is less than the diffraction limit indicated by the black dashed-dotted line and the super-oscillatory criterion indicated by the wine-colored dashed-dotted line over the entire range of *z* considered, resulting in an ultra long super-oscillatory optical hollow needle with a length greater than 11.3*λ*.

### Fabrication method

**Figure 4:**
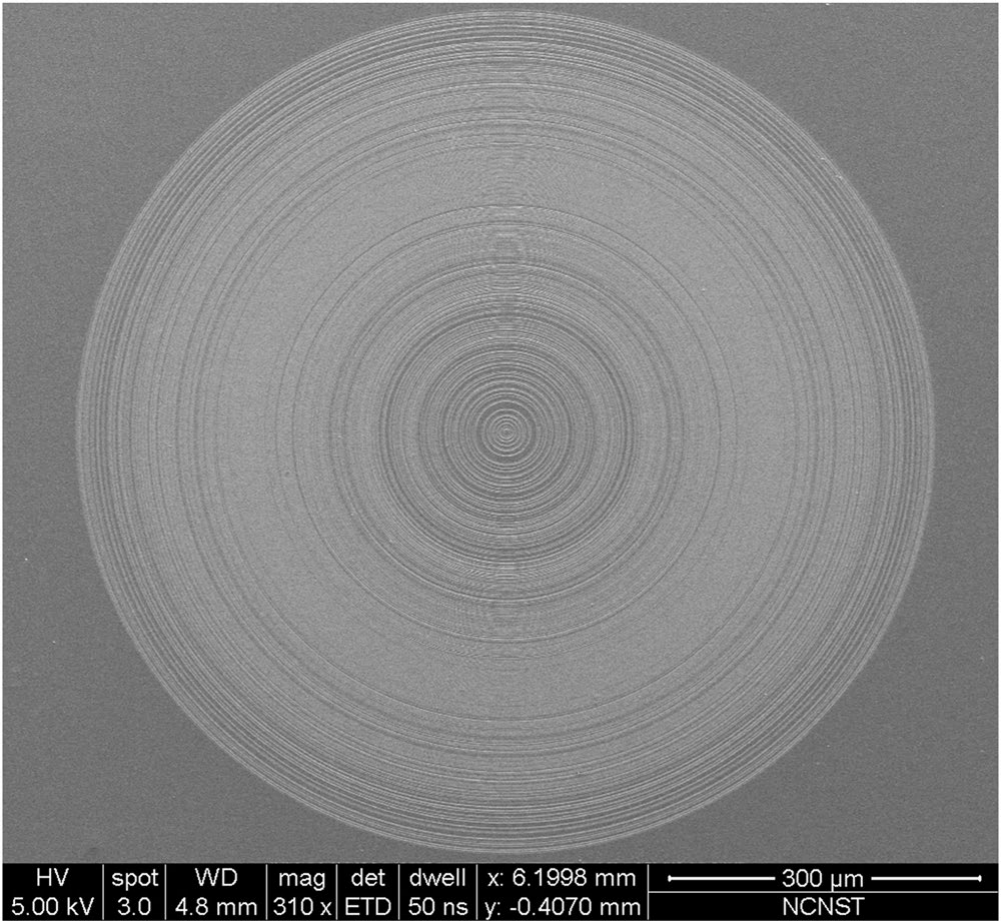
An SEM image of the super-oscillatory binary phase lens with diameter of 822 μm, which consists of 292 Si_3_N_4_ ring belts in the radial direction with smallest ring width of 400 nm.

For lens fabrication, a Si_3_N_4_ layer was first deposited on a 12 × 12 mm^2^ sapphire substrate by plasma-enhanced chemical vapor deposition, which was followed by electron-beam lithography and dry etching to form the Si_3_N_4_ ring belts. The measured refractive index of the Si_3_N_4_ layer was about 1.91, and the Si_3_N_4_ ring belts were fabricated with a 348-nm thickness to achieve a phase difference of π. Figure 4 presents a scanning electron microscopy (SEM) image of the full-size lens with a maximum radius of 411.3 μm.

### Experimental method

**Figure 5:**
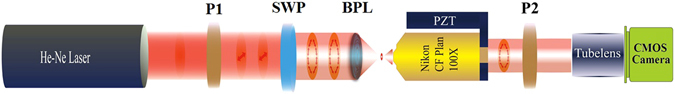
The experimental microscope setup. The laser beam from a He-Ne Laser passes through linear polarizer P1, and is converted to an azimuthally polarized Laguerre-Gaussian wave by S-waveplate SWP. Then, the wave is converted to an optical hollow needle by the super-oscillatory binary-phase lens BPL. A microscope system composed of a Nikon CF plan 100 × objective lens with NA = 0.95, open-loop nano-positioner PZT, tube lens, and CMOS camera is used to obtain the intensity image of the optical field generated by the BPL. The linear polarizer P2 is used only to verify the polarization of the resulting optical hollow needle, and removed otherwise.

The sub-diffraction feature carried by a propagation wave with transverse polarization can be captured with a high NA microscope system^43^. Therefore, the commonly used microscope imaging method^26–28^ shown in Fig. 5 was adopted to obtain the optical intensity distribution of the azimuthally polarized optical hollow needle generated by the super-oscillatory, binary phase lens. In the experimental system, a HeNe laser (HNL 210 L, Thorlabs) operating at *λ* = 632.8 nm is employed as the coherent light source. The beam polarization is controlled by a linear polarizer (P1; IO-2D-633-VLP, Thorlabs). The linearly polarized beam is then converted into an azimuthally polarized Laguerre-Gaussian wave using a commercial S-waveplate (SWP; RPC-632.8-06-188, Workshop of Photonics). The azimuthally polarized beam impinges normally on the planar binary-phase lens (BPL). Then, the optical field intensity is imaged by a microscope system, which consists of a 100 × objective lens (CF Plan 100 × /0.95, Nikon) with NA = 0.95 mounted on an open-loop objective lens nano-positioner (PZT; EO-S1047, Edmund Optics) for conducting the spatial scan in the *z*-direction, a infinity-corrected tube lens (ITL200, Thorlabs), and a CMOS camera with a resolution of 3856 × 2764 and pixel size of 1.67 µm × 1.67 µm (acA3800-14µm, Basler). Neutral density filters are used to adjust the incident beam power. An additional linear polarizer P2 is inserted into the optical path between the objective lens and the tube lens only for verifying the polarization of the optical hollow needle under investigation, and is removed otherwise.

## Results and Discussion

**Figure 6:**
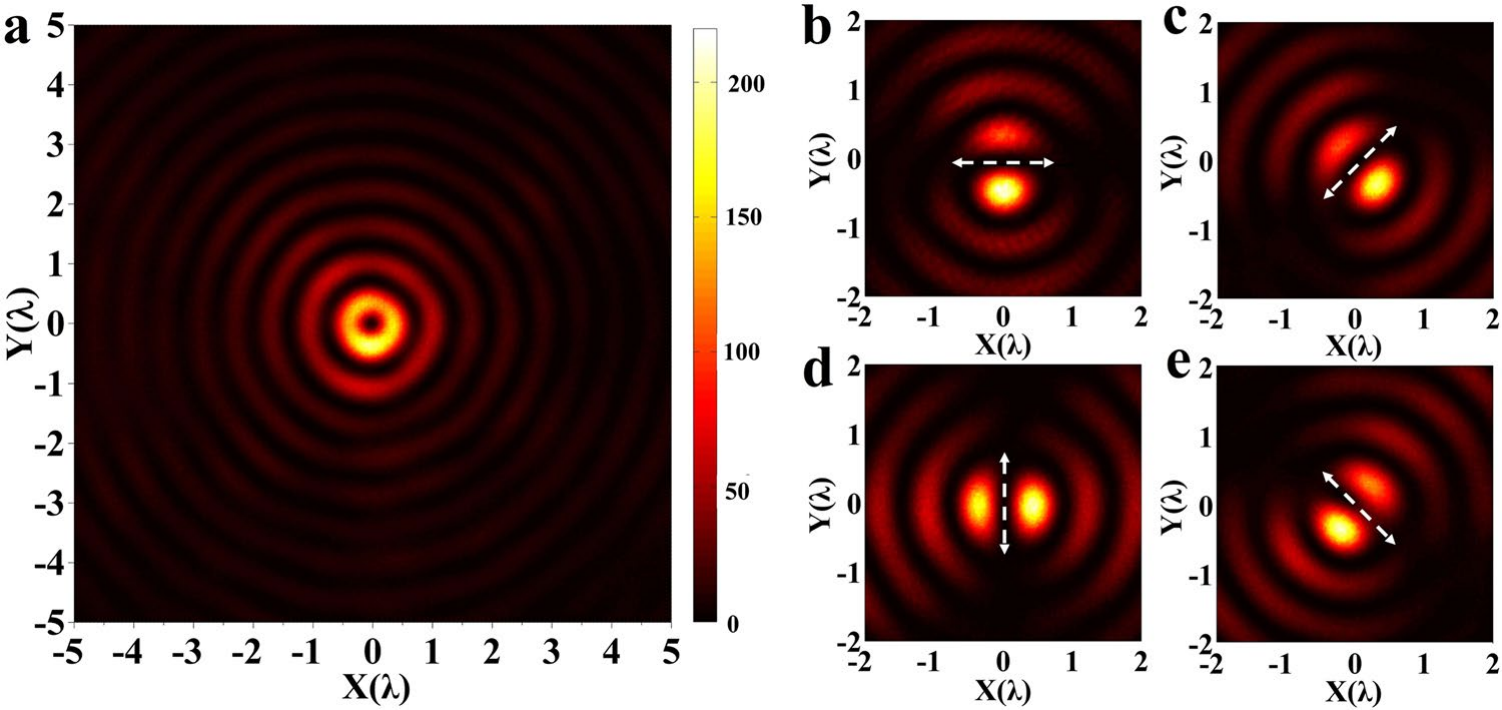
Experimental characterization of the polarization of the optical hollow needle. (**a**) the color map of the in-plane optical intensity on the XY plane at z = 307λ; (**b**)**–**(**e**) the in-plane optical intensity distributions at z = 307λ when linear polarizer P2 (as shown in Fig. 5) is inserted in the optical path in different orientations of 0 deg, 45 deg, 90 deg, and 135 deg, respectively, where the arrows denote the directions of the polarizer. All optical intensity distributions are given in arbitrary unit.

In the experiment, an optical hollow needle was observed around the theoretically determined region. Figure 6(a) presents a color map of the transverse intensity distribution at about *z* = 307*λ*, which clearly exhibits a hollow ring at the center. To verify the azimuthal polarization of the hollow ring, linear polarizer P2 (Fig. 5) was inserted into the optical path, and the resulting images of the hollow ring intensity are presented as color maps in Fig. 6(b)–(e) for the four different polarizer directions indicated by the arrows in the figures, which correspond to the orientations of 0 deg, 45 deg, 90 deg, and 135 deg respectively. These particular intensity distributions provide direct evidence that the optical hollow needle was azimuthally polarized.

**Figure 7:**
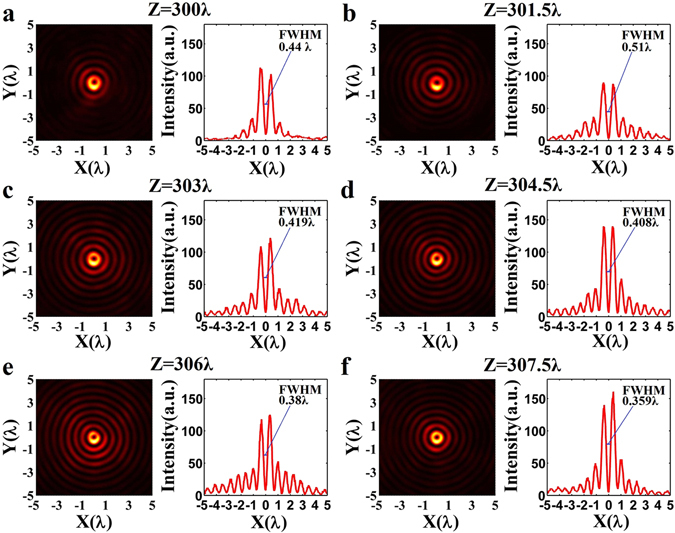
Experimental obtained optical intensity in the designed range of the optical hollow needle. (**a**)**–**(**f**) The color maps of 2-dimentional transverse optical intensity distributions on the XY plane and the transverse optical intensities along the x-axis obtained at different propagation distances of 300*λ*, 301.5*λ*, 303*λ*, 304.5*λ*, 306*λ*, and 307.5*λ*, respectively, where the optical intensity is plotted in arbitrary unit. The corresponding FWHM of the central hollow ring is 0.44*λ*, 0.51*λ*, 0.419*λ*, 0.408*λ*, 0.38*λ*, and 0.359*λ*, respectively.

To understand the hollow needle profile along the optical axis, the transverse optical intensity is obtained with the microscope system at different propagation distances of *z* = 300*λ*, *z* = 301.5*λ*, *z* = 303*λ*, *z* = 304.5*λ*, *z* = 306*λ*, and *z* = 307.5*λ*, respectively. The corresponding experimental results are presented in color maps in Fig. 7(a)–(f). For each color map, the corresponding intensity curve is also plotted with respect to the x-coordinate. As expected, the measured optical intensity distribution on each of these planes shows a clear hollow ring surrounded by several weaker side lobe rings with gradually decaying intensities, which shows good similarity to the numerical simulation results depicted in Fig. 2. It is also seen that, compared with those numerical simulation results, the experimentally obtained intensity distributions are not perfect circularly symmetric. According to our previous work^40^, this skewing of the optical intensity distribution is primarily the result of misalignment in the optics, such as tilted incidence and non-co-axis alignment, owing to difficulties associated with the perfect alignment of the incident beam, the S-waveplate, and the planar lens. To evaluate the transverse dimensions of the hollow ring, an average FWHM was calculated using the FWHM values along the inner diameter of the central ring obtained at ten different directions with a fixed angular step size of 18 degrees. The resulting FWHM averages at *z* = 300*λ*, *z* = 301.5*λ*, *z* = 303*λ*, *z* = 304.5*λ*, *z* = 306*λ*, and *z* = 307.5*λ* are 0.44*λ*, 0.512*λ*, 0.419*λ*, 0.408*λ*, 0.38*λ*, and 0.359*λ*, respectively. We note that the first two FWHM values are less than the diffraction limit 0.551*λ* (0.5*λ*/NA) but greater than the super-oscillatory criterion 0.419*λ* (0.38*λ*/NA), while the final four FWHM values are equal to or less than the super-oscillatory criterion. Obviously, the experimentally obtained transverse size is comparatively larger than the theoretical result given in Fig. 2, because the optical misalignment degrades the symmetry and leads to incensement in the inner transverse size of the central hollow ring. Especially for the generation of such ultra-long optical hollow needle, the alignment is extremely difficult.

**Figure 8:**
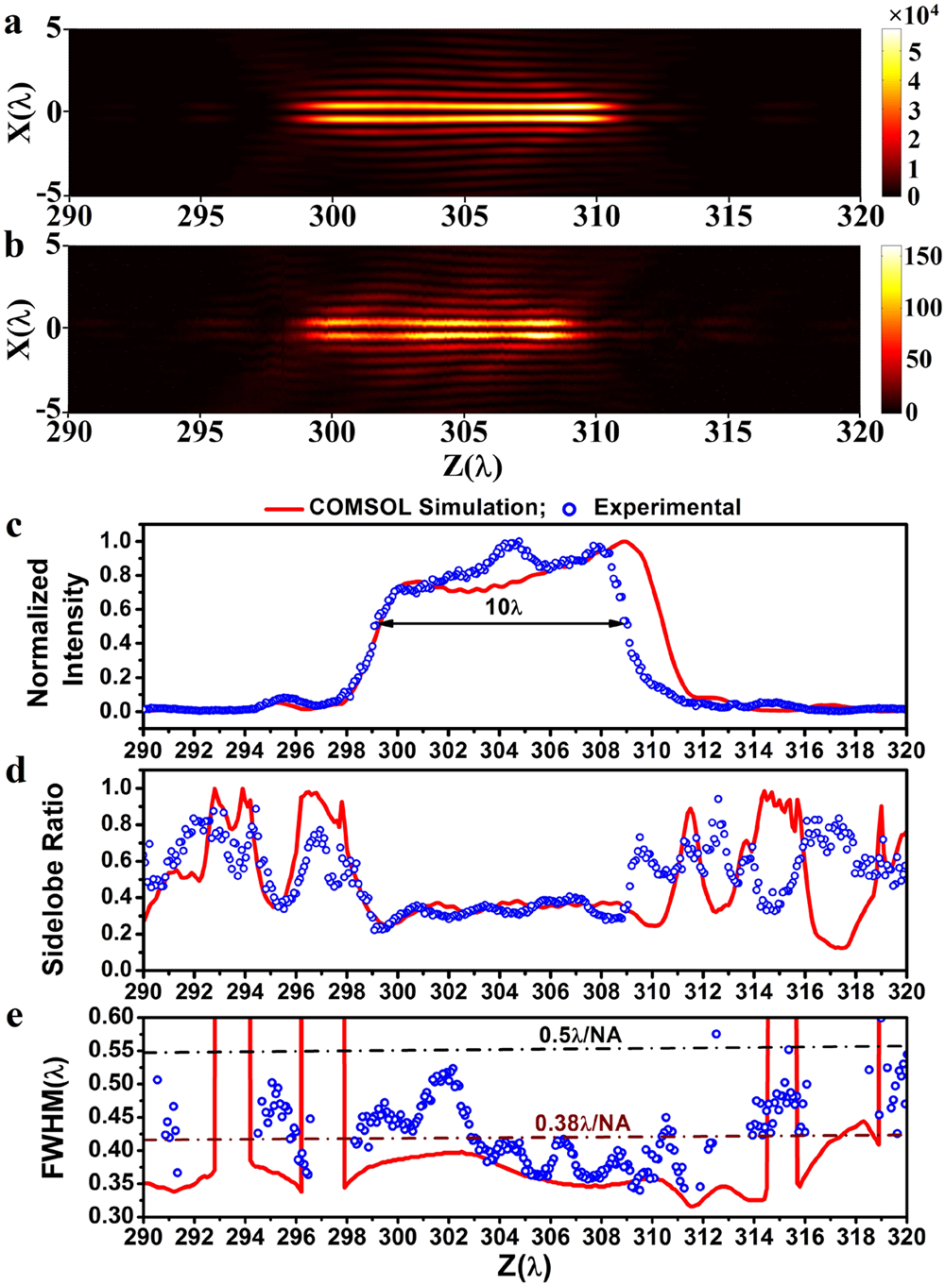
Comparison between COMSOL numerical simulation and experimental results, where the optical intensity, sidelobe ratio, and FWHM are given in arbitrary unit, ratio value, and λ respectively. (**a**) simulation and (**b**) experimental results of the 2-dimentional optical intensity distribution in the XZ propagation plane; (**c**) the normalized central ring peak intensity, (**d**) the sidelobe ratio, and (**e**) the transverse average FWHM distributions along the propagation direction. The black arrow indicated the 10-λ longitudinal FWHM of the experimentally obtained hollow needle. The black and wine-colored dashed-dotted lines indicate the diffraction limit (i.e., 0.5*λ*/NA) and super-oscillatory criterion (i.e., 0.38*λ*/NA), respectively.

To conduct a comprehensive investigation of the properties of the optical hollow needle, optical images of the needle were obtained by the microscope system at equal propagation distance intervals of Δ*z* = 50 nm over a long range of distances from 290*λ* to 320*λ*. For comparison, corresponding COMSOL numerical simulations were conducted, in which ring belts with phase of π are realized with 348 nm-thick Si_3_N_4_ ring belts, which correspond to a phase delay of π with a Si_3_N_4_ refractive index of 1.91 obtained in experiment, compared with the phase change caused by a 348 nm light path in vacuum. Figure 8(a) and (b) present color maps of the simulated and experimentally obtained optical intensity distributions in the XZ propagation plane, respectively. Good agreement is shown between the experimental and simulation results. It is seen that both results show the cross section of a clear long hollow needle structure surrounded by comparatively weaker side lobe rings, and the length of both needles is about 10*λ*. For further comparison, the normalized central ring peak intensity, the side lobe ratio, and the average transverse FWHM obtained by simulation (solid curves) and experimentally (circles) are shown in Fig. 8(c)–(e), respectively. Again, good agreement is observed between simulation and experimental results for these three major parameters. It is found that the longitudinal FWHM of the experimentally obtained optical hollow needle is about 10*λ*, and extends from *z* = 299*λ* to *z* = 309*λ*. It is also noted that the longitudinal length of the hollow needle obtained in the experiment was about 1.5*λ* (1 µm) less than that of the theoretical needle, as observed in Fig. 8(c). This is believed to be caused by an accumulative error in the position of the open-loop nano-positioner that develops during the 380-step scan. The peak intensity distribution show a flat top with a fluctuation less than 30% within the range of the optical hollow needle for both the theoretical and experimental results. As shown in Fig. 8(d), in both the theoretical and experimental results, the sidelobe ratio changes in a similar trend with respect to the propagation distance. Within the range of the optical hollow needle, the sidelobe ratio has a comparatively small value less than 40%. Outside of this range, the sidelobe ratio increases dramatically with a clear decrease in the peak optical intensity. As shown in Fig. 8(e), within the 10λ-long optical hollow needle obtained in the experiment, the transverse FWHM is less than 0.52*λ*, which is less than the diffraction limit of 0.55*λ* (i.e., 0.5*λ*/NA) as indicated by the dark-colored solid line, resulting in a sub-diffraction optical hollow needle with an ultra-long propagation length greater than 10*λ*. However, the measured transverse size is about 0.34*λ*–0.52*λ* in the range z = 298*λ* to z = 310*λ*, and is larger than its theoretical value, which is about 0.316*λ*–0.4*λ* in the range z = 298*λ* to z = 312*λ*. The largest difference in the transverse FWHM is about 0.127*λ* at z = 302.2*λ*. It is also found that the transverse FWHM in the propagation range between *z* = 303*λ* and *z* = 309.5*λ* is less than the super-oscillatory criterion of 0.42*λ* (i.e., 0.38*λ*/NA) as indicated by the wine-color solid line. The smallest transverse FWHM obtained in the experiment was about 0.34*λ* at *z* = 309.5*λ*. Therefore, we have experimentally demonstrated a super-oscillatory optical hollow needle with a length of 6.5*λ* and a sub-diffraction optical hollow needle with a length of 10*λ*. Discrepancies between the simulation and experimental results for the transverse FWHM are primarily caused by the non-symmetrical optical intensity distribution obtained in the experiment as being discussed above. It should be noted that the COMSOL simulation results are a little different from those obtained with equation (2), because the COMSOL simulation takes into account the influence of the lens geometrical thickness, which is ignored in equation (2).

**Figure 9:**
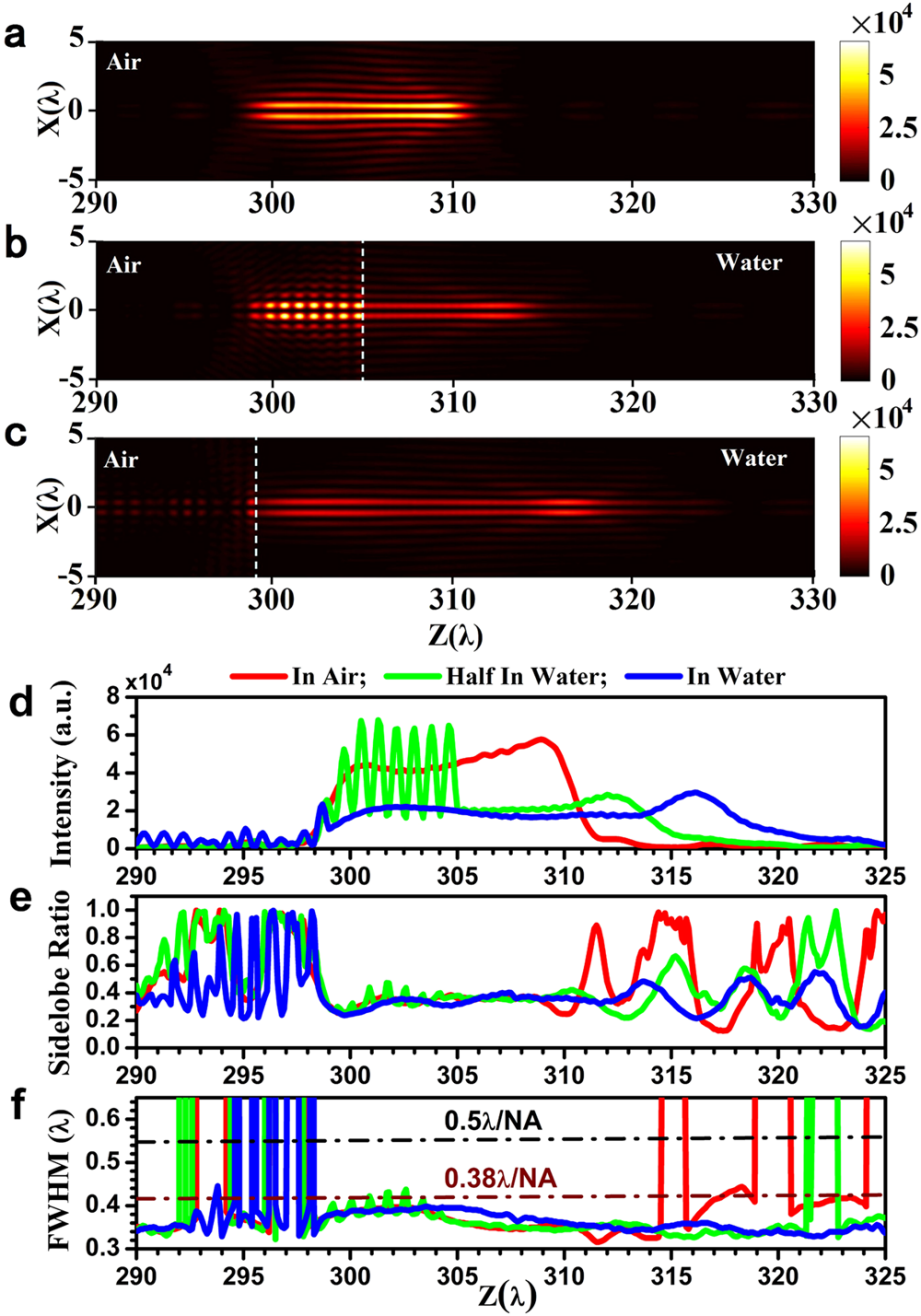
COMSOL numerical simulation results of optical hollow needle performance in air and water. Color maps of the optical hollow needle intensity in the propagation XZ plane (**a**) with the entire needle in air, (**b**) with half the needle in water, and (**c**) with the entire needle in water, where the dashed lines indicate the air-water interface at z = 305λ for (**b**) and z = 299λ for (**c**). The peak intensity (**d**), sidelobe ratio (**e**), and transverse FWHM (**f**) are plotted along the propagation direction for the three different cases of the entire needle in air (red), half the needle in water (green), and the entire needle in water (blue). The optical intensity, sidelobe ratio, and FWHM are given in arbitrary unit, ratio value, and λ respectively. And, the black and wine-colored dashed-dotted lines indicate the diffraction limit and super-oscillatory criterion, respectively.

For practical applications, the proposed optical hollow needle may be required to function in a water environment. To investigate the performance of the super-oscillatory optical hollow needle in water, numerical simulations were also conducted under conditions where half of the optical hollow needle propagates through water from air, and where the full length of the optical hollow needle propagates through water. Figure 9(a)–(c) present color maps of the optical intensity distribution in the propagation plane for the cases where the full length of the optical hollow needle propagates through air, half the length of the needle propagates through water, and the full length of the needle propagates through water, respectively. In these figures, the dashed lines denote the air-water interface. Figure 9(d)–(f) respectively present the central lobe peak intensity, the sidelobe ratio, and the transverse FWHM plotted along the propagation plane from *z* = 290*λ* to *z* = 325*λ*. The peak intensity in water is about half of that when the entire needle is in air. For the later two cases, a clear constructive and deconstructive interference pattern is observed on the air side area near the air-water interface, resulting in an oscillation of optical intensity with a pitch of about 1*λ* on the optic axis, which is attributed to interference between the incident wave and the wave reflected from the air-water interface. Surprisingly, the length of the optical hollow needle in water is enlarged, particularly when the entire needle is immersed in water, which nearly doubles its propagation distance. According to the intensity distribution given in Fig. 9(d), the longitudinal FWHM of the needle is 11.2*λ* (from 299.2*λ* to 310.4*λ*), 15.6*λ* (from 298.6*λ* to 314.2*λ*), and 19.7*λ* (from 298.6*λ* to 318.3*λ*) for the needle solely in air, half in water, and solely in water, respectively, where the maximum intensity in water was employed as the maximum intensity when calculating the longitudinal FWHM for the other two cases. This enlarged non-diffraction propagation distance may be analyzed according to the pure transverse polarization property of the azimuthally polarized beam and the propagation described by Eq. 1. According to electromagnetic theory, the transverse electrical component is continuous on a non-loss dielectric interface. Therefore, the electrical field maintains the same amplitude profile in the nearby regions on both sides of the air-water interface. Due to a shorter wavelength in water, the hollow beam has a smaller Rayleigh range, i.e., *z*_0_ = π*w*_0_^2^ */λ*, resulting in a more slowly increasing beam width *w*(*z*) = *w*_0_[1 + (*z*/*z*_0_)^2^]^1/2^, and, therefore, a slower decay in the optical intensity with respect to the propagation distance. As shown in Fig. 9(e), the sidelobe ratio over the optical hollow needle length range is less than 40% when passing solely through air, the sidelobe ratio is less than 41%, except for an interference peak in the air, when half the needle passes through water, and is less than 41%, except for two small regions around *z* = 313.7*λ* and *z* = 318.5*λ*, when the needle passes solely through water. For all three cases, the transverse FWHM of the optical hollow needle is less than the diffraction limit of 0.5λ/NA, as indicated by the black dashed-dotted line in Fig. 9(f). Moreover, except for those small regions with the strongest constructive interference in air for half the needle passing through water, the transverse FWHM values of the three cases over the longitudinal FWHM region of the needle are less than the super-oscillatory criterion of 0.38λ/NA, as indicated by the wine-colored dashed-dotted line in Fig. 9(f). This indicates a good penetrability of the proposed super-oscillatory optical hollow needle in a water environment without any obvious degradation in the transverse size and sidelobe ratio.

## Conclusions

Although nondiffracting beams have been extensively studied and successfully applied for decades, the experimental generation of their super-oscillatory counterparts is still a challenging and interesting topic for applications such as nanofabrication, optical nanomanipulation, super-resolution microscopy, and nanolithography. Here, we designed and experimentally demonstrated the application of a planar binary-phase dielectric lens to the shaping of a super-oscillatory sub-wavelength optical hollow needle with an azimuthally polarized beam. The focal length was around 300*λ* (190 µm). The resulting optical hollow needle was azimuthally polarized, and its length was greater than 6.5*λ* (4.1 µm at the selected *λ*) for a super-oscillatory transverse size less than 0.42*λ* (265.7 nm), and was longer than 10*λ* (6.3 µm) for a sub-diffraction transverse size less than 0.52*λ* (329.1 nm). The smallest transverse FWHM was about 0.34*λ* (215 nm) in the longitudinal FWHM range of the optical hollow needle. Numerical simulations demonstrated an excellent performance of the proposed optical hollow needle in water with a nearly doubled propagation distance of 19.7*λ* (12.5 µm) and with a super-oscillatory transverse size, making it suitable for practical nanofabrication, optical nanomanipulation, super-resolution microscopy, and nanolithography applications.

## Electronic supplementary material

The structure of the lens
