# Supplementary material for: Planar binary-phase lens for super-oscillatory optical hollow needles
Source: Sci Rep. 2017 Jul 5;7:4697. doi: 10.1038/s41598-017-05060-2 (PMC5498666; doi:10.1038/s41598-017-05060-2)
Supplement: Supplementary file 1 — The structure of the lens [file 41598_2017_5060_MOESM1_ESM.pdf]

Supporting Information

Planar binary-phase lens for super-oscillatory optical hollow needles

Gang Chen[1, †], Zhixiang Wu[1], Anping Yu[1], Kun Zhang[1,2], Jing Wu[1], Luru Dai[2,††], Zhongquan Wen[1,†††], Yinghu He[1,4], Zhihai Zhang[1], Senlin Jiang[1], Changtao Wang[3] & Xiangang Luo[3]

[†, ††, †††]Corresponding Author: E-mail: gchen1@cqu.edu.cn, dai@nanoctr.cn, wenzq@cqu.edu.cn

[1]Key Laboratory of Optoelectronic Technology and Systems (Chongqing University), Ministry of Education, and Key Disciplines Lab of Novel Micro-nano Devices and System Technology, Chongqing University, 173 Shazheng Street, Shapingba, Chongqing 400044, China.
[2]National Center for Nanoscience and Technology, No.11 Zhong Guan CunBei Yi Tiao, Beijing 100190, China.
[3]State Key Laboratory of Optical Technologies on Nano-Fabrication and Micro-Engineering, Institute of Optics and Electronics, Chinese Academy of Science, P. R. Box 350, Chengdu 610209, China.
[4]Department of Physics, Zunyi Normal College, Zunyi 563006, China.

Supplementary

Table S1 the geometrical parameters of the lens, where Ri and Wi are the radius and width of the i-th dielectric ring belt with phase of π, respectively.

i	R_i (μm)	W_i (μm)	i	R_i (μm)	W_i (μm)	i	R_i (μm)	W_i (μm)
**1**	1.1	2.2	**41**	119.4	0.8	**81**	176.4	0.4
**2**	7.6	1.2	**42**	121.6	2.8	**82**	177.2	0.4
**3**	10.8	2	**43**	124	0.4	**83**	178	0.4
**4**	14.6	1.6	**44**	125.2	1.2	**84**	179.2	0.4
**5**	20	2.8	**45**	128	0.4	**85**	180	0.4
**6**	26.6	5.6	**46**	129.2	1.2	**86**	180.8	0.4
**7**	33.8	2.4	**47**	130.8	0.4	**87**	181.8	0.8
**8**	36.6	1.6	**48**	132.8	0.4	**88**	182.8	0.4
**9**	40.2	2.4	**49**	133.6	0.4	**89**	183.6	0.4
**10**	46	2.8	**50**	134.4	0.4	**90**	184.6	0.8
**11**	48.6	0.8	**51**	136.6	0.8	**91**	185.6	0.4
**12**	53.2	2	**52**	138.4	0.4	**92**	186.4	0.4
**13**	56.2	1.6	**53**	139.6	0.4	**93**	187.2	0.4
**14**	58.4	0.4	**54**	140.8	0.4	**94**	188.2	0.8
**15**	61.4	4	**55**	142.4	0.4	**95**	189.2	0.4
**16**	65.6	2	**56**	143.4	0.8	**96**	190	0.4
**17**	70.4	1.2	**57**	145	0.8	**97**	190.8	0.4
**18**	72.8	1.2	**58**	146	0.4	**98**	191.6	0.4
**19**	75	0.8	**59**	147	0.8	**99**	192.8	0.4
**20**	77.2	2	**60**	149	0.8	**100**	193.6	0.4
**21**	81.4	1.6	**61**	150.4	0.4	**101**	194.4	0.4
**22**	84.2	2.4	**62**	151.2	0.4	**102**	195.2	0.4
**23**	86.4	1.2	**63**	152.4	0.4	**103**	196.2	0.8
**24**	89	0.8	**64**	153.4	0.8	**104**	197.2	0.4
**25**	91	0.8	**65**	155.4	1.6	**105**	198	0.4
**26**	92.4	0.4	**66**	157.2	1.2	**106**	198.8	0.4
**27**	94.8	0.4	**67**	159	0.8	**107**	200.4	0.4
**28**	96.6	0.8	**68**	160	0.4	**108**	202.4	0.4
**29**	99	1.6	**69**	160.8	0.4	**109**	203.2	0.4
**30**	101.6	0.4	**70**	161.8	0.8	**110**	204	0.4
**31**	102.4	0.4	**71**	164.8	0.4	**111**	205.4	1.6
**32**	104	1.2	**72**	165.8	0.8	**112**	206.8	0.4
**33**	105.4	0.8	**73**	166.8	0.4	**113**	207.6	0.4
**34**	107.2	1.2	**74**	168.4	1.2	**114**	208.4	0.4
**35**	108.8	0.4	**75**	170.2	1.6	**115**	209.2	0.4
**36**	110.2	1.6	**76**	171.6	0.4	**116**	210	0.4
**37**	111.6	0.4	**77**	172.6	0.8	**117**	210.8	0.4
**38**	114.8	3.6	**78**	173.6	0.4	**118**	211.6	0.4
**39**	117.2	0.4	**79**	174.4	0.4	**119**	212.8	0.4
**40**	118	0.4	**80**	175.4	0.8	**120**	213.6	0.4

Table S1 continued

i	R_i (µm)	W_i (µm)	i	R_i (µm)	W_i (µm)	i	R_i (µm)	W_i (µm)
**121**	214.4	0.4	**161**	248.8	0.4	**201**	283.2	0.4
**122**	215.2	0.4	**162**	249.6	0.4	**202**	284	0.4
**123**	216	0.4	**163**	250.4	0.4	**203**	284.8	0.4
**124**	216.8	0.4	**164**	251.2	0.4	**204**	285.6	0.4
**125**	217.6	0.4	**165**	252	0.4	**205**	286.4	0.4
**126**	218.4	0.4	**166**	252.8	0.4	**206**	287.2	0.4
**127**	219.6	0.4	**167**	253.6	0.4	**207**	288	0.4
**128**	221.6	1.2	**168**	254.4	0.4	**208**	288.8	0.4
**129**	222.8	0.4	**169**	255.2	0.4	**209**	290.4	1.2
**130**	223.6	0.4	**170**	256	0.4	**210**	291.6	0.4
**131**	224.4	0.4	**171**	256.8	0.4	**211**	292.4	0.4
**132**	225.2	0.4	**172**	257.6	0.4	**212**	293.2	0.4
**133**	226	0.4	**173**	258.4	0.4	**213**	294	0.4
**134**	226.8	0.4	**174**	259.2	0.4	**214**	294.8	0.4
**135**	227.6	0.4	**175**	260	0.4	**215**	295.6	0.4
**136**	228.4	0.4	**176**	260.8	0.4	**216**	296.6	0.8
**137**	229.2	0.4	**177**	261.6	0.4	**217**	298	1.2
**138**	230	0.4	**178**	262.4	0.4	**218**	299.6	1.2
**139**	230.8	0.4	**179**	263.2	0.4	**219**	300.8	0.4
**140**	231.8	0.8	**180**	264	0.4	**220**	301.6	0.4
**141**	232.8	0.4	**181**	264.8	0.4	**221**	302.4	0.4
**142**	233.6	0.4	**182**	265.6	0.4	**222**	303.2	0.4
**143**	234.4	0.4	**183**	266.4	0.4	**223**	305.2	1.2
**144**	235.2	0.4	**184**	267.4	0.8	**224**	306.8	0.4
**145**	236	0.4	**185**	268.4	0.4	**225**	307.6	0.4
**146**	236.8	0.4	**186**	269.2	0.4	**226**	308.4	0.4
**147**	237.6	0.4	**187**	270	0.4	**227**	309.2	0.4
**148**	238.4	0.4	**188**	270.8	0.4	**228**	310.2	0.8
**149**	239.2	0.4	**189**	271.6	0.4	**229**	312	0.4
**150**	240	0.4	**190**	272.4	0.4	**230**	312.8	0.4
**151**	240.8	0.4	**191**	273.2	0.4	**231**	313.6	0.4
**152**	241.6	0.4	**192**	274	0.4	**232**	314.8	1.2
**153**	242.4	0.4	**193**	274.8	0.4	**233**	316.6	1.6
**154**	243.2	0.4	**194**	275.6	0.4	**234**	318	0.4
**155**	244	0.4	**195**	276.4	0.4	**235**	318.8	0.4
**156**	244.8	0.4	**196**	277.2	0.4	**236**	319.6	0.4
**157**	245.6	0.4	**197**	278.4	1.2	**237**	320.4	0.4
**158**	246.4	0.4	**198**	279.6	0.4	**238**	321.4	0.8
**159**	247.2	0.4	**199**	280.6	0.8	**239**	322.4	0.4
**160**	248	0.4	**200**	282.4	0.4	**240**	323.2	0.4

Table S1 continued

i	R_i (μm)	W_i (μm)	i	R_i (μm)	W_i (μm)	i	R_i (μm)	W_i (μm)
**241**	324	0.4	**259**	343	1.6	**277**	370.4	0.4
**242**	324.8	0.4	**260**	344.4	0.4	**278**	373.2	2.8
**243**	325.8	0.8	**261**	345.2	0.4	**279**	375.6	0.4
**244**	326.8	0.4	**262**	346	0.4	**280**	377	1.6
**245**	327.6	0.4	**263**	347	0.8	**281**	379.2	0.4
**246**	328.4	0.4	**264**	348	0.4	**282**	383.2	3.6
**247**	329.2	0.4	**265**	348.8	0.4	**283**	388.2	3.2
**248**	330.2	0.8	**266**	349.8	0.8	**284**	390.4	0.4
**249**	332	0.4	**267**	351.6	0.4	**285**	391.2	0.4
**250**	332.8	0.4	**268**	352.8	1.2	**286**	393.4	3.2
**251**	333.6	0.4	**269**	354	0.4	**287**	398.6	2.4
**252**	335	1.6	**270**	355.6	1.2	**288**	400.6	0.8
**253**	336.4	0.4	**271**	357.8	0.8	**289**	402.8	1.2
**254**	337.2	0.4	**272**	360.2	2.4	**290**	405.2	2
**255**	338	0.4	**273**	362.2	0.8	**291**	408	0.4
**256**	338.8	0.4	**274**	363.6	1.2	**292**	410.4	1.2
**257**	340.8	0.4	**275**	367	1.6			
**258**	341.6	0.4	**276**	369.6	0.4			

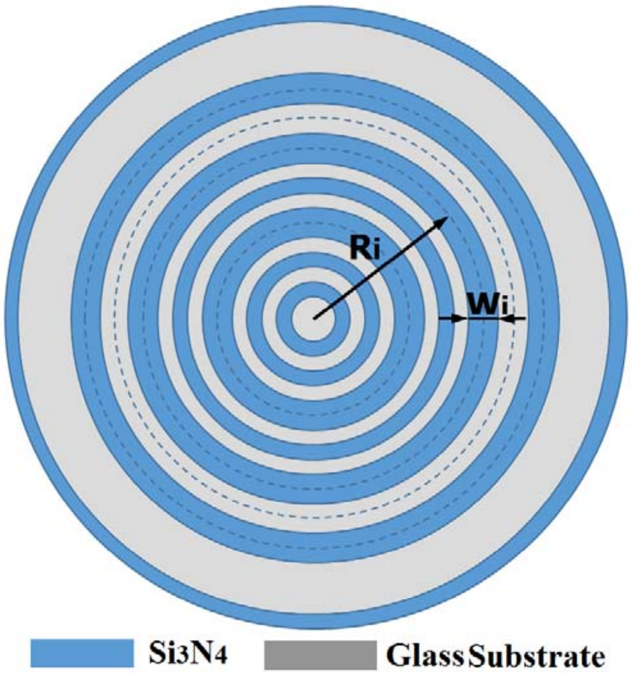

Figure S1 the structure of the lens: the lens consists of 292 Si_3N_4 concentric ring belts. The radius and width of the i-th Si_3N_4 ring belt are R_i and W_i, respectively. The refractive index of Si_3N_4 is 1.91, and thickness of the Si_3N_4 ring belts is 348 nm, which corresponds to a relative phase delay of π, compared with the phase change caused by a 348 nm light path in vacuum.
